# Detection of cell-type-specific differentially methylated regions in epigenome-wide association studies

**DOI:** 10.1093/bioinformatics/btaf243

**Published:** 2025-07-15

**Authors:** Ruofan Jia, Yingying Wei

**Affiliations:** Department of Statistics, The Chinese University of Hong Kong, Shatin, NT, Hong Kong SAR, China; Department of Statistics, The Chinese University of Hong Kong, Shatin, NT, Hong Kong SAR, China

## Abstract

**Motivation:**

DNA methylation at cytosine–phosphate–guanine (CpG) sites is one of the most important epigenetic markers. Therefore, epidemiologists are interested in investigating DNA methylation in large cohorts through epigenome-wide association studies (EWAS). However, the observed EWAS data are bulk data with signals aggregated from distinct cell types. Deconvolution of cell-type-specific signals from EWAS data is challenging because phenotypes can affect both cell-type proportions and cell-type-specific methylation levels. Recently, there has been active research on detecting cell-type-specific risk CpG sites for EWAS data. However, existing methods all assume that the methylation levels of different CpG sites are independent and perform association detection for each CpG site separately. Although these methods significantly improve the detection at the aggregated-level—identifying a CpG site as a risk CpG site as long as it is associated with the phenotype in any cell type, they have low power in detecting cell-type-specific associations for EWAS with typical sample sizes.

**Results:**

Here, we develop a new method, Fine-scale inference for Differentially Methylated Regions (FineDMR), to borrow strengths of nearby CpG sites to improve the cell-type-specific association detection. Via a Bayesian hierarchical model built upon Gaussian process functional regression, FineDMR takes advantage of the spatial dependencies between CpG sites. FineDMR can provide cell-type-specific association detection as well as output subject-specific and cell-type-specific methylation profiles for each subject. Simulation studies and real data analysis show that FineDMR substantially improves the power in detecting cell-type-specific associations for EWAS data.

**Availability and implementation:**

FineDMR is freely available at https://github.com/JiaRuofan/Detection-of-Cell-type-specific-DMRs-in-EWAS.

## 1 Introduction

Although single-cell sequencing technologies have become prevalent, the high cost still prevents researchers to assay hundreds or even thousands of subjects via single-cell sequencing. As a result, epigenome-wide association studies (EWAS) that investigate the associations between DNA methylation at cytosine–phosphate–guanine (CpG) sites and phenotypes still have to rely on bulk data. Since EWAS data are signals aggregated from distinct cell types, it is crucial to deconvolute the cell-type-specific signals from the observed aggregated-level data. However, inferring cell-type-specific associations from the observed aggregated-level data is very challenging due to the huge number of parameters that need to be estimated.

To infer the cell-type-specific associations for *q* phenotypes for an EWAS dataset that assayed *G* CpG sites for tissues containing *K* cell types, we need to estimate G×K parameters for the baseline DNA methylation levels for *K* cell types, q×G×K parameters for the phenotype effects and (K−1)×n parameters for each subject’s cell-type proportions. The total number of (GK+GqK+(K−1)n) parameters can be on a similar scale as the total number of *Gn* observed values. As a result, cell-type-specific association detection suffers from low statistical power when the sample size *n* is small or moderate. Given that DNA methylation is one of the most important epigenetic markers and has been associated with many phenotypes of interest, association detection methods with high statistical power are urgently needed.

From the early days of EWAS, people have been aware that the variations in cellular compositions can confound the association detection, and ignoring such heterogeneity can lead to a huge number of spurious associations (McGregor *et al.* 2016). Therefore, to adjust the confounding factor, people first tried to add cell-type proportions or surrogates learned for cell-type proportions as covariates to linear regressions and regress the DNA methylation levels on both the phenotypes and the cell-type proportions (Leek and Storey 2007, Zou *et al.* 2014). As pointed out by McGregor *et al.* (2016), although these methods can in general control type-one error, they can suffer from extremely low power, as low as 3.6% as illustrated by McGregor *et al.* (2016).

One major issue with these early approaches is that they treated cell-type proportions as additive effects. However, cell-type proportions impose multiplicative effects so that the products of the phenotypes and the cell-type proportions need to be added to the regression for association detection. Based on this observation, HIRE (Luo *et al.* 2019), TOAST (Li *et al.* 2019), TCA (Rahmani *et al.* 2019), and CellDMC (Zheng *et al.* 2018) were independently proposed to model cell-type proportions as multiplicative effects. As a result, these methods not only substantially improved the association detection but also enabled the identification of cell-type-specific associations. Although these methods have greatly improved the power in detecting the risk CpG site at the aggregated level—identifying a CpG site as a risk CpG site as long as it is associated with the phenotype in any one of all the cell types, their power in detecting cell-type-specific risk CpG sites are still very low given a typical EWAS that assays hundreds of subjects. As a result, association detection methods that can provide cell-type-specific inference with high statistical power is still lacking.

One of the reasons why the current state-of-the-art cell-type-specific association detection methods lose power is that they all ignore the spatial correlations between CpG sites and detect associations separately for each individual CpG site. However, it has been found that methylation levels of nearby CpG sites are indeed highly correlated (Jaffe *et al.* 2012, Hebestreit *et al.* 2013, Aryee *et al.* 2014). As a result, a large literature has been devoted to the identification of differentially methylated regions (DMRs) (Jaffe *et al.* 2012, Pedersen *et al.* 2012, Hebestreit *et al.* 2013, Sofer *et al.* 2013, Aryee *et al.* 2014, Feng *et al.* 2014). However, all of these existing methods ignore the fact that EWAS data are signals aggregated from different cell types and cellular heterogeneity can confound the association detection, and they only declare associations at the aggregated level.

Therefore, it is desirable to borrow the strengths of nearby CpG sites to improve the power for detecting cell-type-specific associations for EWAS data. Here, we propose a method, FineDMR, to perform Fine-scale inference for the aggregated-level EWAS data and identify the DMRs that are associated with phenotypes for each individual cell type. Via a Bayesian hierarchical model based on the Gaussian process functional regression model, FineDMR leverages the spatial correlations between nearby CpG sites. It can simultaneously (i) infer baseline methylation profile for each cell type, (ii) detect cell-type-specific associations for each phenotype in each cell type, (iii) learn the cell-type proportions of each subject directly from the data, and (iv) estimate subject-specific cell-type-specific methylation levels for each subject and each cell type. Both simulation studies and real data analysis show that FineDMR substantially outperforms state-of-the-art methods in identifying cell-type-specific associations, especially when the sample size is small to moderate.

## 2 Materials and methods

### 2.1 Model

In an EWAS study that has assayed *n* subjects, for a given subject *i*, we observe the aggregated-level DNA methylation levels $O_{i}={\left(O_{1i},\ldots ,O_{Gi}\right)}^{T}$ of a total of *G* CpG sites and sample *i*’s *q* phenotypes $X_{i}={\left[X_{i1},\ldots ,X_{iq}\right]}^{T}$, such as age, gender, smoking history, and disease status. Because the methylation levels of nearby CpG sites are highly correlated, we can view $O_{i}$ as observations of a smooth function $O_{i}(t)$ at genomic locations $\left(t_{1},\ldots ,t_{G}\right)$, where $t_{g}$ is the position of the *g*th CpG site on the genome.

However, a sample collected from an EWAS study consists of distinct cell types. Let us denote the cellular compositions of sample *i* as $p_{i}=(p_{i1},\ldots ,p_{iK})$ and its subject-specific methylation profile for cell type *k* as $Y_{ik}(t)$. Consequently, $O_{i}(t)=\sum_{k=1}^{K}p_{ik}Y_{ik}(t)+\tau_{i}(t)$, where the residual $\tau_{i}(t)$ follows a Gaussian process $\tau_{i}(t)∼GP[a(t),S(\cdot ,\cdot ;v,w)]$ with the mean function a(t)=0 for any *t* and the covariance function $S(t_{1},t_{2};v,w)=v\cdot \;\text{exp}\;\{-\frac{1}{2}w{\left(t_{1}-t_{2}\right)}^{2}\}$. The scaling parameter *v* corresponds to the variation of a typical function, and the bandwidth parameter *w* controls the function’s smoothness. The model thus borrows strengths from nearby CpG sites and lets nearby CpG sites have higher correlations. Our goal is to understand the association between $Y_{ik}(t)$ and the phenotypes $X_{ij}$ for each cell type *k* given only the aggregated-level data $O=(O_{1},\ldots ,O_{n})$ from the *n* samples.

Therefore, to associate phenotypes to methylation levels, we assume that the subject-specific cell-type-specific methylation levels $Y_{ik}(t)$ are affected by the phenotypes via a functional regression model with a functional response and scalar covariates: $Y_{ik}(t)=m_{k}(t)+\sum_{j=1}^{q}\beta_{kj}(t)X_{ij}+\epsilon_{ik}(t),\epsilon_{ik}(t)∼N(0,\sigma_{\epsilon }^{2})$. Here, $\epsilon_{ik}(t)$s at different genomic locations are independent, whereas the baseline cell-type-specific methylation levels $m_{k}(t)$ and the effects of phenotypes on cell-type-specific methylation levels $\beta_{kj}(t)$ vary across CpG sites. We further use the B-splines to model $m_{k}(t)$ and $\beta_{kj}(t)$ with basis functions denoted as $\Phi (t)={\left(\Phi_{1}(t),\ldots ,\Phi_{D}(t)\right)}^{T}$ so that $m_{k}(t)=C_{\mathrm{k0}}\Phi (t)$ and $\beta_{kj}(t)=C_{\mathrm{kj}}\Phi (t)$, where $C_{\mathrm{kj}}={\left[C_{kj1},\ldots ,C_{\mathrm{kjD}}\right]}^{T}$.

**Figure 1.**
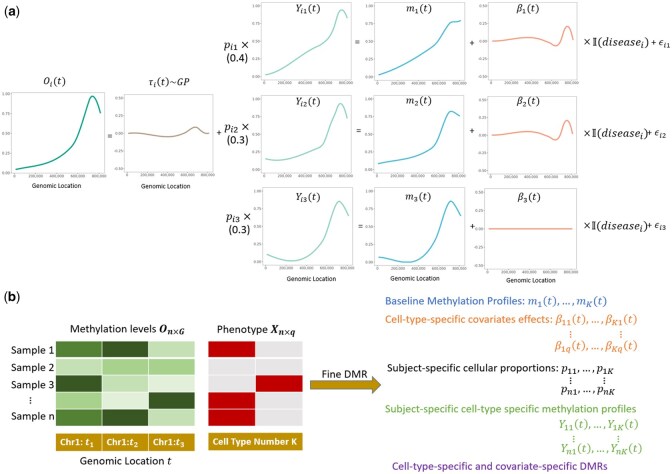
A simple illustration of FineDMR with three cell types (K=3) and one phenotype (disease status: q=1). (a) The data generating process assumed by FineDMR. $O_{i}(t)$s are the observed methylation levels and can be viewed as a function of genomic location *t*. They are signals aggregated from the cell-type-specific methylation levels $Y_{ik}(t)$s with cellular proportions $p_{i}$ and an error term $\tau_{i}(t)$ following the Gaussian process. In addition to the cell-type-specific baseline methylation levels $m_{k}(t)$s, the cell-type-specific methylation levels $Y_{ik}(t)$s are affected by the cell-type-specific associations $\beta_{k}(t)$s of the covariates. (b) The input and output of FineDMR. The input includes the observed methylation levels of *G* CpG sites of *n* samples, the *q* phenotypes of *n* samples, the genomic location of *G* CpG sites and the number of cell types *K*. The output includes the baseline methylation levels for *K* cell types, the cell-type-specific covariates effects, the subject-specific cellular proportions, subject-specific and cell-type-specific methylation profiles and the cell-type-specific and covariate-specific DMRs.

Collectively, our proposed FineDMR model can be summarized as follows (Fig. 1): 


$$\begin{array}{c} O_{i}(t)=\sum_{k=1}^{K}p_{ik}Y_{ik}(t)+\tau_{i}(t);\\ Y_{ik}(t)=m_{k}(t)+\sum_{j=1}^{q}\beta_{kj}(t)X_{ij}+\epsilon_{ik}(t),\epsilon_{ik}(t)∼N(0,\sigma_{\epsilon }^{2});\\ \tau_{i}(t)∼GP[0,S(\cdot ,\cdot ;v,w)],\text{cov}(\tau_{i}(t_{1}),\tau_{i}(t_{2}))=S(t_{1},t_{2};v,w);\\ m_{k}(t)=C_{\mathrm{k0}}\Phi (t);\beta_{kj}(t)=C_{\mathrm{kj}}\Phi (t); \end{array}$$

for i=1,…,n,k=1,…,K,j=1,…,q.

Consequently, the observed data W consist of the aggregated-level methylation values O and the phenotype data $X=(X_{1},\ldots ,X_{n})$, and the unknown parameters that need to be estimated, denoted by Ω, include $\left\{C_{k}={\left(C_{k0}^{T},\ldots ,C_{kq}^{T}\right)}^{T},k=1,\ldots ,K\right\}$, $\left\{p_{i},i=1,\ldots ,n\right\}$, $\sigma_{\epsilon }^{2}$, and θ=(v,w).

We estimate the parameters under the Bayesian framework and impose noninformative priors for the parameters. Specifically, we impose flat priors for the spline coefficients $p(C_{\mathrm{kjl}})\propto 1,k=1,\ldots ,K,j=0,\ldots ,q,l=1,\ldots ,D$ and for cellular compositions $P(p_{i})\propto 1$. Moreover, following Shi and Choi (2011), we impose prior distributions for the parameters of the Gaussian process as follows: $w^{-1}∼Ga(\alpha_{w},\alpha_{w}/\mu_{w})$, $\log(v)∼N(\mu_{v},\sigma_{v}^{2})$, and ${\left[\sigma_{\epsilon }^{2}\right]}^{-1}∼Ga(\alpha_{\sigma^{2}},\beta_{\sigma^{2}})$. Note that $E(w^{-1})=\mu_{w}$ and $\text{var}(w^{-1})=\frac{\mu_{w}^{2}}{\alpha_{w}}$. Therefore, if $\tau_{i}(t)$ is believed to be constant, a large value should be assigned to $\mu_{w}$. Meanwhile, small values of $\alpha_{w}$, $\alpha_{\sigma^{2}}$, and $\beta_{\sigma^{2}}$ and a large value of $\sigma_{v}^{2}$ produce vague priors.

As it is difficult to directly optimize the posterior distribution, we develop a generalized expectation-maximization (GEM) algorithm (Dempster *et al.* 1977) to estimate the parameters by augmenting the missing data $Y_{ik}(t)$ for each subject and each cell type. GEMs are more computationally efficient than Markov chain Monte Carlo algorithms. The outline of the GEM algorithm is as follows: (i) in the *E*-step, we calculate the *Q*-function $Q(\Omega |\Omega^{\left(t\right)})=E(\text{log}\;p(\Omega |W,Y)|W;\Omega^{\left(t\right)})$. Noteworthy, the conditional distribution of $Y_{ik}=(Y_{ik}(t_{1}),\ldots ,Y_{ik}(t_{g}))|W,\Omega^{\left(t\right)}$ also follows a multivariate normal distribution, so the *E*-step can be calculated analytically. (ii) Update $C_{k}^{\left(t+1\right)}$s by maximizing $Q(\Omega |\Omega^{\left(t\right)})$. (iii) Update $p_{i}^{\left(t+1\right)}$s by maximizing $Q(\Omega |\Omega^{\left(t\right)})$ given $C_{k}=C_{k}^{\left(t+1\right)},k=1,\ldots ,K$ using quadratic programming. (iv) Update $\sigma_{\epsilon }^{2}$ by maximizing $Q(\Omega |\Omega^{\left(t\right)})$ given $C_{k}=C_{k}^{\left(t+1\right)},k=1,\ldots ,K$ and $p_{i}=p_{i}^{\left(t+1\right)},i=1,\ldots ,n$. (v) Update $\theta^{\left(t+1\right)}$ by maximizing $Q(\Omega |\Omega^{\left(t\right)})$ given $C_{k}=C_{k}^{\left(t+1\right)},k=1,\ldots ,K$, $p_{i}=p_{i}^{\left(t+1\right)},i=1,\ldots ,n$ and $\sigma_{\epsilon }^{2}={\left[\sigma_{\epsilon }^{2}\right]}^{\left(t+1\right)}$ using gradient descent. We list the details of the GEM algorithm in Supplementary Section S4.

One challenge in the computation is that calculating the conditional expectation and conditional covariance of $Y_{ik}|W,\Omega^{\left(t\right)}$ involves the inversion of a G×G matrix, which is often the case for models built upon Gaussian processes. Fortunately, because CpG sites are usually clustered and $S(t_{1},t_{2};v,w)=v\cdot \;\text{exp}\;\{-\frac{1}{2}w{\left(t_{1}-t_{2}\right)}^{2}\}$ decays exponentially with the distance between two locations $t_{1}$ and $t_{2}$, we can divide the whole genome into blocks based on the distance. Consequently, both the coefficients for the splines basis $C_{k}$s and the conditional covariance of $Y_{ik}|W,\Omega^{\left(t\right)}$ enjoy the property of being block-wise diagonal. Consequently, we can parallelize the updates of parameters for different blocks to speed up the computation, and we allow different blocks to contain different numbers of CpG sites.

With the GEM algorithm, we can obtain the posterior mode of Ω, and the association effects can be estimated accordingly by ${\hat{\beta }}_{kj}(t_{g})={\hat{C}}_{kj}\Phi (t_{g})$. As cell-type-specific DMRs for phenotype *j* and cell type *k* are defined as the contiguous intervals $R_{\mathrm{jkh}},h=1,\ldots ,H_{jk}$ for which $\beta_{kj}(t)=0$ for all $t\in R_{\mathrm{jkh}}$, we need to perform hypothesis testing. According to Bayesian asymptotic theory, when the sample size is large, the posterior mode will converge to the maximum likelihood estimator (Vaart 2000). Therefore, we construct Wald-type test statistics which enjoy analytic distributions to avoid the heavy computational burden caused by approaches such as permutation test and bootstrap. Specifically, noticing that the marginal distribution of $O_{i}∼N({\left([1,X_{i}^{T}]\sum_{k=1}^{K}p_{ik}^{\left(t\right)}C_{k}\Phi^{T}\right)}^{T},\Gamma_{i})$ where $\Gamma_{i}=S(v,w)+\sum_{k=1}^{K}p_{ik}^{2}\sigma_{\epsilon }^{2}I$. Here, S(v,w) is a matrix whose (e,f) element is $S(t_{e},t_{f};v,w)$ and *I* is a G×G identity matrix. Thus, by viewing $C_{k}$ as parameters of the linear regression that regress $O_{ig}$ on $X_{ij}\times p_{i}\times \Phi (t)$ and plugging in $\hat{v}$, $\hat{w}$, ${\hat{\sigma }}_{\epsilon }^{2}$ and $\hat{p}$, we can use the weighted least square to derive the variance–covariance matrix of $\text{vec}\hat{\left(C\right)}$ by ${\left(U^{T}\mathrm{VU}\right)}^{-1}$, where vec(C) = $\left\{C_{\mathrm{kmd}},k=1,\ldots K,m=0,\ldots q,d=1,\ldots D\right\}$,

$U=\left[\begin{array}{cccccc} p_{11}\Phi & \cdots & p_{11}X_{1q}\Phi & p_{12}\Phi & \cdots & p_{1K}X_{1q}\Phi \\ \vdots & \vdots & \vdots & & & \\ p_{n1}\Phi & \cdots & p_{n1}X_{nq}\Phi & p_{n2}\Phi & \cdots & p_{nK}X_{nq}\Phi \end{array}\right]$ , $V=\left[\begin{array}{ccc} \Gamma_{1}^{-1} & & \\ & \ddots & \\ & & \Gamma_{n}^{-1} \end{array}\right]$. The variance of ${\hat{\beta }}_{kj}(t_{g})$ can then be estimated by: $\text{var}({\hat{\beta }}_{kj}(t_{g}))=\sum_{l_{1}}^{D}(\Phi_{l_{1}}{\left(t_{g}\right)}^{2}\text{var}(C_{kjl_{1}})+2\sum_{l_{1}<l_{2}\leq D}\Phi_{l_{1}}(t_{g})\Phi_{l_{2}}(t_{g})\text{cov}(C_{kjl_{1}},C_{kjl_{2}}))$. Thus, according to Bayesian asymptotic theory, the test statistic $z_{kj}(t_{g})={\hat{\beta }}_{kj}(t_{g})/\sqrt{\text{var}({\hat{\beta }}_{kj}(t_{g}))}$ follows the standard normal distribution under the null hypothesis, and the corresponding *p*-value becomes $p_{kj}(t_{g})=(1-\phi (|z_{kj}(t_{g})|))\times 2$, where ϕ(x) is the cumulative distribution function of the standard normal distribution. Following Luo *et al.* (2019), we use Bonferroni correction to adjust multiple hypothesis testing. For a given family-wise error rate (FWER) α, we reject $H_{0}:\beta_{kj}(t_{g})=0$ if and only if $p_{kj}(t_{g})<\alpha /(G\times K\times q)$. We then generate candidate regions $R_{\mathrm{jkh}}$s by collecting contiguous CpG sites that have rejected the null hypotheses, following the similar approach taken by Hebestreit *et al.* (2013). By doing so, we are able to directly compare the true positive rates (TPRs) and false positive rates (FPRs) with state-of-the-art methods for detecting cell-type-specific risk CpG sites, to the best of our knowledge no methods for detecting cell-type-specific DMRs are available so far.

## 3 Results

### 3.1 Simulation

We conducted extensive simulation studies to evaluate the performance of FineDMR and compared it with the state-of-the-art methods for detecting cell-type-specific CpG sites for EWAS data—HIRE, TOAST, TCA, and CellDMC. As TOAST, TCA, and CellDMC require the input of the cellular compositions, we first ran the algorithm of Houseman *et al.* (2016) to obtain the cellular compositions following the common practice.

We generated datasets in which the observed DNA methylation levels are signals aggregated from K=6 cell types, and we associated each sample with a diseased or normal status $X_{i}$ sampled from a Bernoulli distribution Bernoulli(0.6). We let the cellular compositions depend on the disease status and drew $p_{i}=(p_{i1},\ldots ,p_{iK})$ from a Dirichlet distribution $\mathrm{Dirichlet}(2,2,2,2,2+x_{i})$. To mimic genomic locations of CpG sites assayed by typical methylation arrays used in EWAS, we took the genomic locations of the first G=10 000 CpG sites of Chromosome 1 assayed by Infinium HumanMethylation EPIC array, which can be split into 134 blocks with each block containing 50–100 CpG sites (see Supplementary Section S1). The genomic locations of the CpG sites within the same block are clustered, whereas the distances between CpG sites from different blocks are large enough so that their correlations are ignorable. We adopted cubic splines for the basis functions Φ(t). Φ(t) were generated independently for each block, and the degree of freedom was set as 0.1×blocksize+3 for each block. Then, to generate the baseline DNA methylation levels $m_{k}(t)=C_{\mathrm{k0}}\Phi (t)$, we sampled $C_{k0d}$ from Beta(1,8) with probability 0.9 and from Beta(3,1) with probability 0.1. In particular, we also considered the cell lineage. After generating the baseline DNA methylation levels $m_{1}(t)$ for cell type 1, in order to make the methylation profiles of cell types 2 and 3 similar to that of cell type 1, we sampled the cell-type-specific DNA methylation levels of a specific genomic location $m_{2}(t)$ and $m_{3}(t)$ for cell types 2 and 3 from $N(m_{1}(t),0.05)$.

We investigated two scenarios: (i) the true null case in which there are no associations between methylation levels and the phenotype so that $\beta_{k1}(t)=0$ for any cell type *k* and any genomic location *t* and (ii) the true alternative case in which there exist contiguous intervals $R_{1kh}$ with $\beta_{k1}(t)\neq 0$ for $t\in R_{1kh}$. We allowed both DMRs with positive effects $\beta_{k1}(t)>0$ and DMRs with negative effects $\beta_{k1}(t)<0$. Once again, we considered the cell lineage when generating the association effects: if a genomic region is a DMR for disease status in cell type 1, it has the probability of 0.6 to be also differentially methylated between cases and controls with the same direction of signals in cell types 2 and 3.

**Figure 2.**
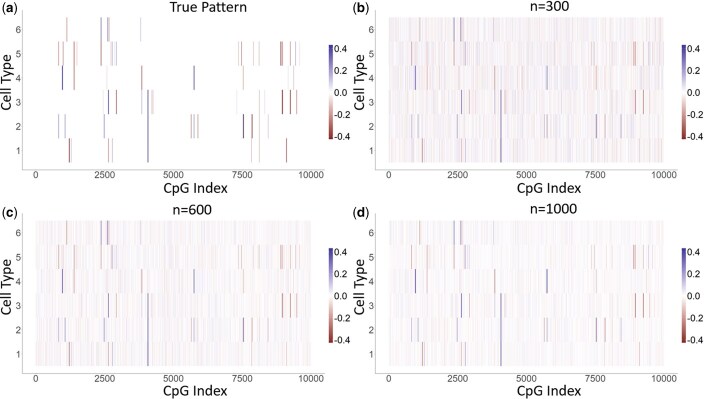
The true and the estimated association patterns for simulation studies under the true alternative case. (a) The underlying true pattern. The learned effect sizes by FineDMR when (b) *n* = 300, (c) *n* = 600, and (d) *n* = 1000. FineDMR is able to learn the effect sizes well.

Among the generated DMRs, 80% have fewer than 20 CpG sites and 20% consist of 20–40 CpG sites. We let each DMR have equal probability to belong to one of the six cell types. Instead of generating the effect sizes using splines following our model assumption, we sampled the effect sizes independently from N(0.4,0.01) for 20% DMRs, from N(−0.4,0.01) for 20% DMRs, from N(0.2,0.01) for 40% DMRs, and from N(−0.2,0.01) for 40% DMRs in order to evaluate FineDMR’s performance in the model misspecification scenario. We chose the effect size according to Jaffe *et al.* (2012) in which the average effect size in a DMR at the bulk level is around 0.2 (see Fig. 1 of Jaffe *et al.* 2012). Thus, suppose a DMR is associated with the disease status in *k* of the total *K* cell types with the same directions of signals and the cellular proportions are roughly even, then the effect sizes at the cell-type-specific level should be around 0.2/(k/K). In our simulation setting, a DMR occurs in at most three of the six cell types. Therefore, at the cell-type-specific level, we inflated the bulk-level effect size and set it around (0.2/(3/6))=0.4. As a result, if a DMR is associated with the disease status in three out of the six cells because of cell lineage, the effect size of the DMR at the bulk level is approximately 0.2; when the DMR is associated with the disease status in only one of the six cell types, the effect size of the DMR at the bulk level is approximately 0.07. Compared to the 40% DMRs with around 0.4 or −0.4 cell-type-specific effect sizes, the 60% DMRs with around 0.2 or −0.2 cell-type-specific effect sizes have even weaker signals. The assumed underlying true association patterns are shown in Fig. 2a.

We set the variance of the residual for cell-type-specific methylation levels $\sigma_{\epsilon }^{2}=0.01$ and the parameters for the Gaussian process v=0.01 and w=2. We also varied the sample size *n* among 300, 600, and 1000 for each simulation setting to study the impact of the sample size.

We compared each method’s ability in controlling false positives and power to detect risk-CpG sites. As all the other methods were developed for detecting risk CpG sites instead of DMRs, we compared the TPRs and FPRs at the scale of CpG site. The TPRs and the FPRs were compared for both the detection at the aggregated-level—a CpG site is said to be a risk CpG site as long as it is significant in any one of the *K* cell types—and the cell-type-specific level. We set the FWER α=0.01 for all methods.

**Figure 3.**
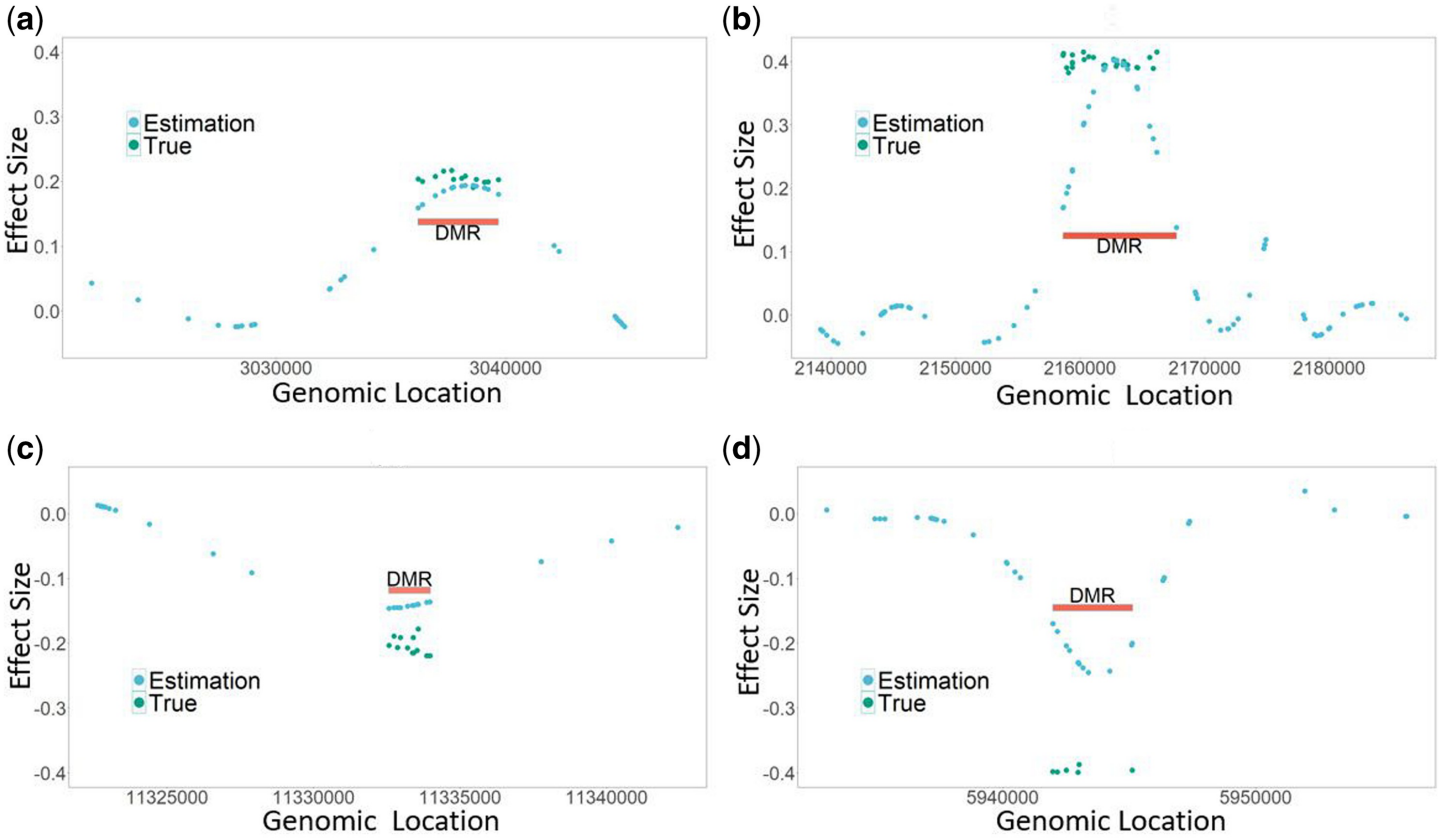
Examples of DMRs in (a) cell type 6, (b) cell type 3, (c) cell type 5, and (d) cell type 2 for the simulation study. The blue points denote the effect sizes learned by FineDMR for the true alternative simulation setting when n=300 that have associations with the disease status. The red bars indicate the identified DMRs. FineDMR can detect the DMRs regardless of the sizes of the effects (the left column with the absolute values of the effect sizes around 0.2 and the right column with the absolute values of the effect sizes around 0.4) and the directions of the signals (the top row for positive effects and the bottom row for negative effects).

**Figure 4.**
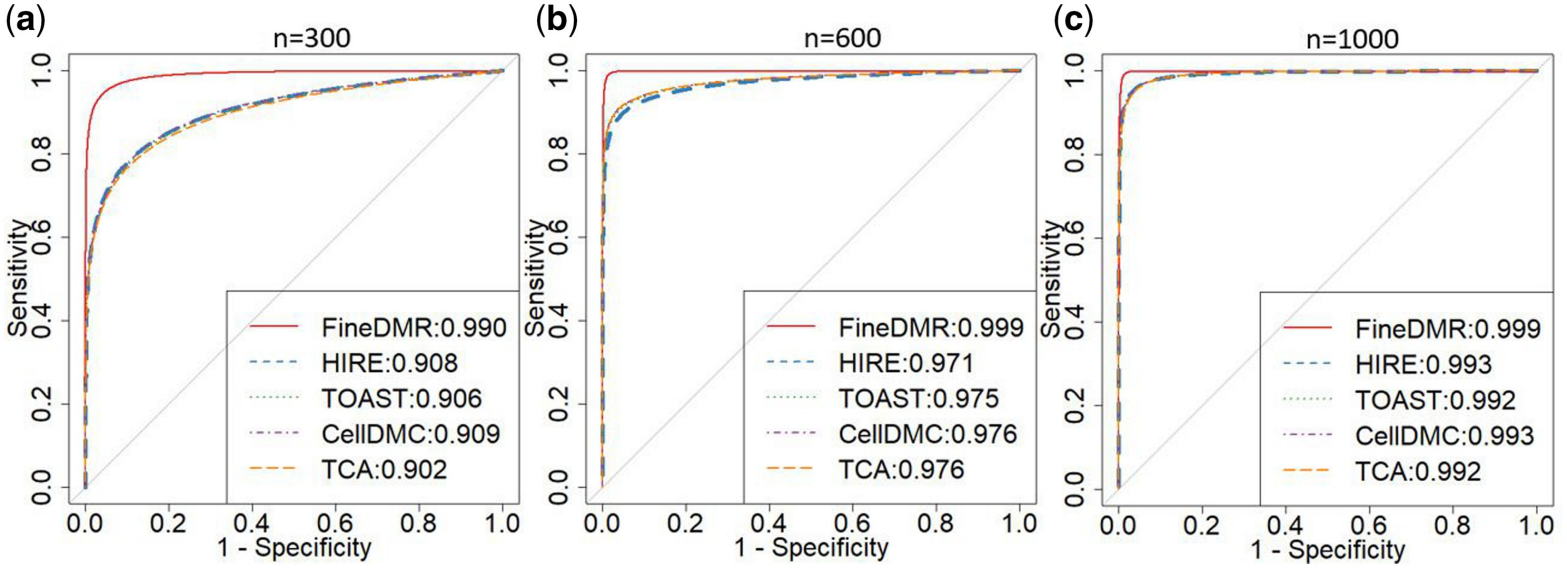
ROC curves of FineDMR and the compared methods for simulations under the true alternative case when (a) *n* = 300, (b) *n* = 600, and (c) *n* = 1 000.

FineDMR is able to learn the parameters accurately even when the sample size is as small as n=300 (Figs 1–3). FineDMR successfully identified all the underlying DMRs for each cell type. In comparison, under the true null, all methods are able to control the FPRs very well: none of them are greater than 0.005 (Table 1). However, for HIRE, TOAST, TCA, and CellDMC, even with the sample size being n=1000, when the TPRs at the aggregated-level are relatively high, they lack power in detecting cell-type-specific risk CpG sites, with CellDMC achieving 43.6% and being the highest. When the sample size decreases to n=600, a typical size of EWAS, the power in detecting cell-type-specific risk CpG sites by the existing methods further drops below 30%. In contrast, FineDMR is able to identify 83.4% cell-type-specific associations. For a small sample size n=300, the state-of-the-art methods suffer from low TPRs for even the association detection at the aggregated-level as reported in literature (Luo *et al.* 2019). In contrast, with the sample size n=300, FineDMR is still able to identify 82.1% cell-type-specific risk CpG sites, thus substantially improving the power for association detection. The receiver operating characteristic (ROC) curves, whose performances are not affected by the threshold of significance level, also confirm the advantage of FineDMR, especially when the sample size is small (Fig. 4).

**Table 1.** Performance of FineDMR and the compared methods in simulation studies.

	*G*	*n*	*K*		FineDMR	HIRE	TOAST	TCA	CellDMC
True null	10 000	300	6	FPR (aggregated)	0.001	0	0	0	0
				FPR (cell-type-specific)	0.0002	0	0.001	0	0
	10 000	600	6	FPR (aggregated)	0	0	0	0	0
				FPR (cell-type-specific)	0	0	0	0	0
	10 000	1000	6	FPR (aggregated)	0	0	0	0	0
				FPR (cell-type-specific)	0	0	0	0	0
True alternative	10 000	300	6	TPR (aggregated)	0.851	0.07	0.065	0.062	0.066
				FPR (aggregated)	0.01	0	0	0	0
				TPR (cell-type-specific)	0.821	0.049	0.045	0.043	0.046
				FPR (cell-type-specific)	0.003	0	0	0	0
	10 000	600	6	TPR (aggregated)	0.889	0.3	0.319	0.33	0.333
				FPR (aggregated)	0.003	0	0	0	0
				TPR (cell-type-specific)	0.834	0.245	0.258	0.264	0.268
				FPR (cell-type-specific)	0.0005	0	0	0	0
	10 000	1000	6	TPR (aggregated)	0.991	0.513	0.493	0.53	0.527
				FPR (aggregated)	0.01	0	0	0	0
				TPR (cell-type-specific)	0.984	0.426	0.412	0.433	0.436
				FPR (cell-type-specific)	0.005	0.00002	0	0.00002	0.00003

### 3.2 Real data analysis

We compared FineDMR with the state-of-the-art methods on two famous benchmarking EWAS datasets—the rheumatoid arthritis (RA) dataset (Liu *et al.* 2013) and the GALA II blood methylation dataset (Pino-Yanes *et al.* 2015). As an illustration, we focused on Chromosome 1, the longest chromosome. Details of the real data preprocessing can be found in Supplementary Section S3.

The RA dataset profiled the DNA methylation levels for whole blood from 354 RA patients and 335 normal subjects. In addition to the RA status, other covariates include gender, smoking history, and age. People with different smoking histories were categorized into four groups: “never,” “current,” “ex,” and “occasional” smokers. Therefore, we treated smoking history as a nominal variable and chose “never” as the baseline. Therefore, with the three dummy variables for smoking history, there are in total six covariates. Following the previous analysis of the RA dataset, we set the number of cell types K=6 (Rahmani *et al.* 2016, Luo *et al.* 2019) and applied all the compared methods. As TOAST, TCA, and CellDMC require the input of cellular compositions, we first applied the algorithm of Houseman *et al.* (2016) to learn the cellular compositions. Unfortunately, TCA and CellDMC failed to run with the input cellular compositions. Therefore, we just report the results obtained by FineDMR, HIRE, and TOAST. Despite batch effects and biological variabilities, all six cell types learned by FineDMR can be matched to known cell type references (see Supplementary Section S2 for cell-type matching protocol). Specifically, cell type 1 was matched to CD56+ natural killer cell (CD56+ NK); cell types 2 and 6 were annotated as neutrophils; cell type 3 was aligned to CD14+ monocytes; cell type 4 was matched to CD4+ T cell; and cell type 5 was annotated as CD8+ T cell.

**Figure 5.**
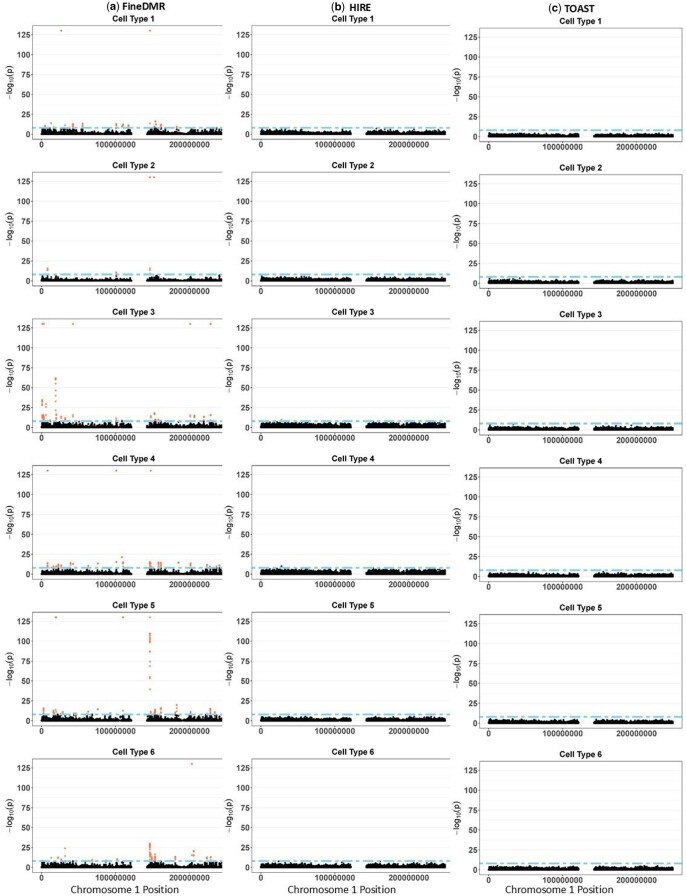
Manhattan plots of the −log_10_(*p*-value) for the cell-type-specific associations with the RA status in the RA dataset by (a) FineDMR, (b) HIRE, and (c) TOAST. Cell type 1 was annotated as CD56+ natural killer cell (CD56+ NK); cell types 2 and 6 were annotated as neutrophils; cell type 3 was annotated as CD14+ monocytes; cell type 4 was annotated as CD4+ T cell; and cell type 5 was annotated as CD8+ T cell.

At the FWER α=0.01, for the RA status, FineDMR detected 20, 4, 33, 29, 31, and 23 DMRs of RA status in cell types 1–6, respectively, which corresponds to a total of 143, 38, 231, 222, 267, and 166 cell-type-specific risk CpG sites. Thus, cell types 3 (CD14+ monocytes), 4 (CD4+ T cell), and 5 (CD8+ T cell) have the largest numbers of DMRs associated with RA status, confirming the critical roles of monocytes and T cells for RA development (McGarry *et al.* 2021, Chemin and Malmström 2023). The details for the other covariates can be found in Table 2. In contrast, as shown in Table 2 and the Manhattan plots in Fig. 5, the number of risk CpG sites called by HIRE and TOAST is much smaller, similar to the simulation studies. Thus, FineDMR is much more powerful in detecting cell-type-specific associations.

**Table 2.** The number of DMRs and risk CpG sites identified by FineDMR and the compared methods in two real datasets.

RA dataset ^a^							
	Cell-type-specific						Agg
Cell type	1	2	3	4	5	6	
DMRs							
RA status	20	4	33	29	31	23	
Sex	27	7	20	18	16	25	
Age	35	9	42	49	39	25	
CurrSmoke	22	5	37	25	38	17	
ExSmoke	20	2	32	21	22	9	
OccasSmoke	9	2	22	28	33	8	
FineDMR							
RA status	143	38	231	222	267	166	692
Sex	239	78	172	138	125	186	923
Age	264	79	281	384	347	208	615
CurrSmoke	185	72	269	169	335	102	736
ExSmoke	167	32	256	146	179	62	551
OccasSmoke	78	24	160	228	286	85	541
HIRE							
RA status	0	0	1	0	0	0	1
Sex	2	0	0	0	1	0	2
Age	0	0	0	1	1	1	3
CurrSmoke	0	0	0	1	0	0	1
ExSmoke	0	0	0	0	0	0	0
OccasSmoke	0	0	0	0	0	0	0
TOAST							
RA status	0	0	0	0	0	0	0
Sex	0	14	1	0	0	0	14
Age	0	40	0	0	0	0	40
CurrSmoke	0	0	0	0	0	0	0
ExSmoke	0	0	0	0	0	0	0
OccasSmoke	0	0	0	0	0	0	0

GALA II blood methylation dataset ^b^							
	Cell-type-specific						Agg
Cell type	1	2	3	4	5	6	
DMRs							
Sex	42	182	70	186	132	709	
MixedLatino	32	67	32	57	43	44	
PuertoRican	71	111	167	123	132	755	
OtherLatino	65	56	53	58	81	60	
FineDMR							
Sex	406	1371	580	1548	1088	5683	7564
MixedLatino	258	521	223	454	349	348	1106
PuertoRican	511	904	1399	980	959	6442	8032
OtherLatino	561	445	376	513	566	430	1548
HIRE							
Sex	7	3	7	5	5	362	369
MixedLatino	2	7	1	3	4	7	13
PuertoRican	1111	767	116	99	670	837	2560
OtherLatino	16	7	0	1	0	5	28
TOAST							
Sex	60	20	0	21	3	0	83
MixedLatino	3	5	3	4	3	5	12
PuertoRican	5	121	287	47	2	56	418
OtherLatino	1	0	0	13	3	2	17

“Agg” stands for aggregated-level.

a In RA dataset, cell type 1 was annotated as CD56+ natural killer cell (CD56+ NK); cell types 2 and 6 were annotated as neutrophils; cell type 3 was annotated as CD14+ monocytes; cell type 4 was annotated as CD4+ T cell; and cell type 5 was annotated as CD8+ T cell.

b In GALA II dataset, cell types 1, 3, and 4 were annotated as neutrophils; cell type 5 was annotated as CD14+ monocytes; and cell type 6 was annotated as CD4+ T cell. The cell type 2 cannot be aligned to the references.

**Figure 6.**
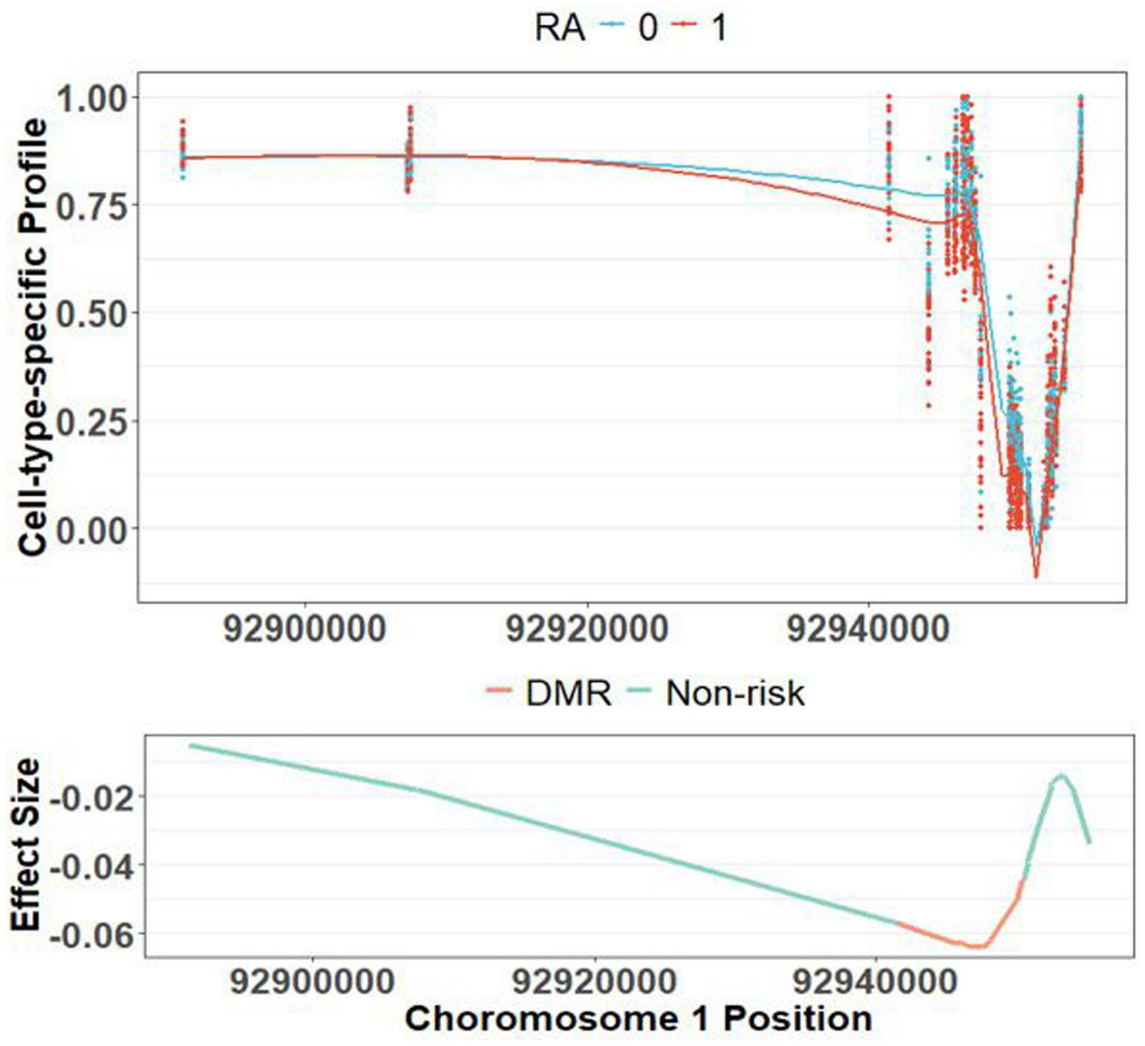
Pattern of a DMR associated with the RA status identified by FineDMR in cell type 5 (annotated as CD8+ T cell). The upper panel shows the subject-specific and cell-type-specific methylation levels learned by FineDMR of 50 randomly selected samples, with controls denoted by blue and RA patients by red. The lower panel shows the learned effect size $\beta_{51}(t)$.

Figure 6 plots the subject-specific cell-type-specific methylation levels learned by FineDMR of 50 randomly selected samples around a DMR associated with RA status (Chromosome 1: 92944306–92950348) for cell type 5 (annotated as CD8+ T cell). The closest genes to this DMR include CD1A, CD1B, CD1C, and CD1E. When gene set enrichment analysis was applied to the genes of the risk CpG sites associated with the RA status learned by FineDMR in cell type 5, three KEGG pathways were found when controlling the false discovery rate at 0.01 (Table 3). CD1A, CD1B, CD1C, and CD1E all belong to these three pathways. Noteworthy, both hematopoietic stem cell function and tight junction are well known to be closely related to RA (Moore *et al.* 2003, Mucientes *et al.* 2024).

**Table 3.** KEGG pathways enriched among risk CpG sites identified by FineDMR in the two real datasets.

Pathway name	Covariates	Cell type	*p*.adjust
RA dataset^a^			

Hematopoietic cell lineage	RA Status	5	0.00001
Amoebiasis	RA Status	5	0.00001
Tight junction	RA Status	5	0.00002
Hematopoietic cell lineage	Smoking: Current	2	0.00001
Amoebiasis	Smoking: Current	2	0.00001
Tight junction	Smoking: Current	2	0.00004
Glycosaminoglycan biosynthesis—heparan sulfate/heparin	Smoking: Occasional	4	0.006
Hematopoietic cell lineage	Smoking: Occasional	5	0.001
Amoebiasis	Smoking: Occasional	5	0.001
Tight junction	Smoking: Occasional	5	0.003
Cobalamin transport and metabolism	Smoking: Occasional	5	0.004
Glycosaminoglycan biosynthesis—heparan sulfate/heparin	Smoking: Occasional	5	0.005
Starch and sucrose metabolism	Smoking: Occasional	2	0.005

GALA II blood methylation dataset^b^			

Olfactory transduction	Sex	5	0
Metabolism of xenobiotics by cytochrome P450	Sex	1	0.001
Drug metabolism—cytochrome P450	Mixed Latino	3	0.01

a In RA dataset, cell type 2 was annotated as neutrophils; cell type 4 was annotated as CD4+ T cell; and cell type 5 was annotated as CD8+ T cell.

b In GALA II dataset, cell types 1 and 3 were annotated as neutrophils; and cell type 5 was annotated as CD14+ monocytes.

The GALA II blood methylation dataset is an EWAS that assayed 573 Latino children. Each child belongs to one of the following four groups: “Mexican,” “Mixed Latino,” “Puerto Rican,” and “Other Latino.” Except for ethnicity, gender information is also available for each sample. We treated ethnicity as a nominal variable and chose “Mexican” as the baseline, thus creating three dummy variables. Therefore, with gender, there are in total four covariates. Following the previous analysis of the same dataset, we set the number of cell types K=6 when running all the compared methods (Rahmani *et al.* 2016, Luo *et al.* 2019). Once again, TCA and CellDMC failed to run with the input cellular compositions, so we only report the results obtained by FineDMR, HIRE, and TOAST. According to the cell-type-specific DNA methylation profiles for each cell type learned by FineDMR, five out of the six cell types can be matched to known blood cell references: cell types 1, 3, and 4 were matched to neutrophils; cell type 5 was aligned to CD14+ monocytes; cell type 6 was annotated as CD4+ T cell, whereas cell type 2 cannot be aligned to the references.

**Figure 7.**
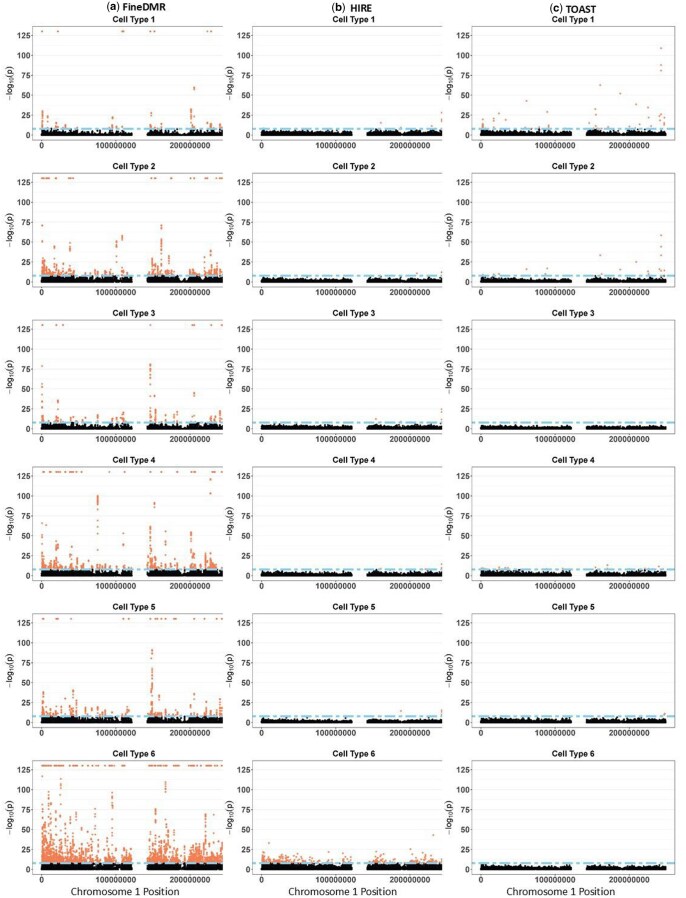
Manhattan plots of the −log_10_(*p*-value) for the cell-type-specific associations with sex in the GALA II dataset by (a) FineDMR, (b) HIRE, and (c) TOAST. Cell types 1, 3, and 4 were annotated as neutrophils; cell type 5 was annotated as CD14+ monocytes; and cell type 6 was annotated as CD4+ T cell. The cell type 2 cannot be aligned to the references.

At FWER α=0.01, FineDMR identified 42, 182, 70, 186, 132, and 709 DMRs associated with sex in cell types 1–6, respectively, which corresponds to a total of 406, 1371, 580, 1548, 1088, and 5683 cell-type-specific risk CpG sites. The cell-type-specific association detection results are listed in Table 2. Once again, both Table 2 and the Manhattan plots in Fig. 7 show that FineDMR is much more powerful in identifying cell-type-specific risk CpG sites both at the aggregated-level and cell-type-specific level.

**Figure 8.**
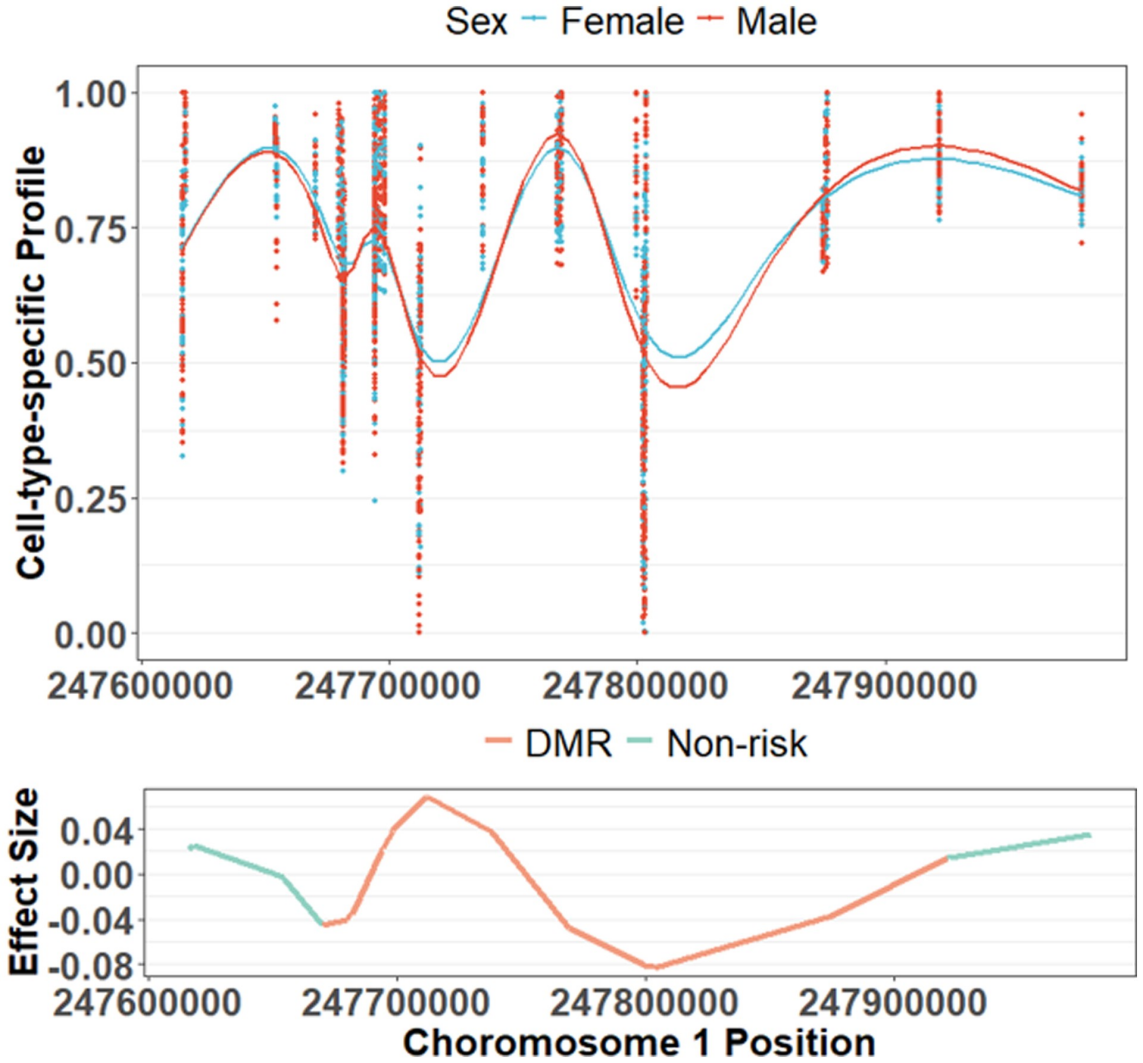
Pattern of a DMR associated with sex identified by FineDMR in cell type 5 (annotated as CD14+ monocytes) for the GALA II dataset. The upper panel shows the subject-specific and cell-type-specific methylation levels learned by FineDMR of 50 randomly selected samples, with females denoted by blue and males by red. The lower panel shows the learned effect size $\beta_{51}(t)$.

Figure 8 plots the subject-specific cell-type-specific methylation levels learned by FineDMR of 50 randomly selected samples around a DMR associated with sex (Chromosome 1: 247670081–247876443) for cell type 5 (annotated as CD14+ monocytes). The closest genes to this DMR include OR2W5, GCSAML-AS1, OR2C3, GCSAML, OR2G3, OR13G1, and OR6F1. Noteworthy, the KEGG pathway olfactory transduction is enriched for risk CpG sites associated with sex in cell type 5, and OR2C3, OR2G3, OR13G1, and OR6F1 all belong to the olfactory transduction pathway, which is closely related to sex (Oliveira-Pinto *et al.* 2014). Thus, FineDMR offers biologically meaningful discoveries.

## 4 Discussion

We propose a Bayesian hierarchical model, FineDMR, which leverages the spatial correlations between CpG sites to make it easier to identify moderate but contiguous signals. As a result, FineDMR substantially improves the detection of cell-type-specific associations compared to the state-of-the-art methods for detecting cell-type-specific risk CpG sites for EWAS data.

Currently, FineDMR requires the input of cell type number *K* as TOAST, TCA, and CellDMC do. However, in principle, when *K* is unknown *a priori*, it can be learned according to the penalized Bayesian information criterion as HIRE does (Luo *et al.* 2019). In addition, FineDMR also asks for the input of the degree of freedom for the B-splines. However, simulation studies show that FineDMR is robust to the misspecification of the degree of freedom, even if we varied it from the true assumed value of blocksize/10+3 to blocksize/5+3 and blocksize/15+3, respectively (see Supplementary Table S1).

One of FineDMR’s advantages is that it does not require the input of cell-type proportions for each subject but learns them from the data. In contrast, TOAST, TCA, and CellDMC all require cell-type proportions as input to their algorithms. Therefore, they usually first estimate the cell-type proportions by either regressing the methylation data on a set of reference methylation profiles or using reference-free methods to learn the cellular compositions of each sample via quadratic programming. Since these reference-based and reference-free methods all assume that the cell-type-specific methylation profiles are the same for all the subjects and not affected by the phenotypes, the estimation of cell-type proportions is inaccurate. As a result, in practice, TCA and CellDMC failed to run on the two real datasets we analyzed with the cell-type proportions estimated *a priori*.

In this article, we adjust the multiple hypothesis testing according to the total number of CpG sites to allow direct comparison with the state-of-the-art methods. Nevertheless, if *p*-values for each DMR is needed, we can further derive it according to the theory of functional data analysis (Ramsay and Silverman 2005), which we will incorporate in the future. In an analogy, we are now providing point-wise confidence intervals yet with the most conservative adjustment for multiple hypothesis testing using the Bonferroni correction instead of confidence bands, whereas the confidence band of a function may also be of interest. FineDMR outputs both *p*-values and cell-type-specific effect sizes so that users can use their domain knowledge to determine whether a statistically significant association indicates a biologically meaningful difference according to the effect size.

We provide the C code of FineDMR freely available and will soon further wrap it into an R package. We envision that FineDMR will greatly facilitate the analysis of EWAS data and enable many new discoveries in the field of epigenetics.

## Supplementary Material

btaf243_Supplementary_Data

## Data Availability

The simulated data is available at https://github.com/JiaRuofan/Detection-of-Cell-type-specific-DMRs-in-EWAS. The two real data sets analyzed are publicly available at GEO with accession number GSE42861 and GSE77716.
