# Supplementary material for: Detection of cell-type-specific differentially methylated regions in epigenome-wide association studies
Source: Bioinformatics. 2025 Jul 15;41(Suppl 1):i502–12. doi: 10.1093/bioinformatics/btaf243 (PMC12261422; doi:10.1093/bioinformatics/btaf243)
Supplement: btaf243_Supplementary_Data [file btaf243_supplementary_data.zip › btaf243_Supplementary_Data/FineDMR_supplementary.pdf]

Supplementary Materials for Detection of Cell-type-specific Differentially Methylated Regions in Epigenome-Wide Association Studies

Ruofan Jia[1] and Yingying Wei *[1]

[1] *Department of Statistics, The Chinese University of Hong Kong*

S1 Block Assignment

We use a greedy search algorithm to assign the CpG sites to each block. We first set the maximum length of a block as len_{max} and a minimum length of a block as len_{min}. Suppose that all the first $I_s - 1$ CpG sites have already been allocated to blocks, for CpG site I_s, we calculate the distances between neighboring CpG sites for CpG sites $I_s + len_{min} - 1, \cdots, I_s + len_{max}$ and denote the distances as $d_{I_s+len_{min}}, \cdots, d_{I_s+len_{max}}$. We then choose $I_{s+1} = argmax(d_{I_s+len_{min}}, \cdots, d_{I_s+len_{max}})$ as the left end point of the next block and identify CpG sites I_s to $I_{s+1} - 1$ as one block. We repeat this process until the number of remining CpG sites is smaller than len_{max}.

We re-scaled the genomic locations with 0.01 to faciliate the computation and minimize the risk of numeric overflow in computation.

S2 Cell Type Matching Protocol

We aligned the learned cell types by mapping the cell-type-specific methylation levels estimated by FineDMR to the existing reference methylation levels following [1]. Suppose we have the existing reference methylation levels for A cell types of g_s CpG sites, denoted by $\boldsymbol{\eta}_1, \ldots, \boldsymbol{\eta}_A$. Let $\hat{\boldsymbol{Y}}_k$ denote a vector of length g_s of which the gth element is the median of the estimated profiles $\hat{Y}_{ik}(t_g)$ across samples. Then, for each estimated cell type k, we calculated the cosine similarity ρ_{ka} between $\hat{\boldsymbol{Y}}_k$ and $\boldsymbol{\eta}_a$. Cosine similarity is choosen since it helps to protect against batch effects [1]. We matched the etimated cell type k with existing reference cell type a if ρ_{ka} ranked the highest among $\rho_{k1}, \ldots, \rho_{kA}$ and exceeded 0.9.

The reference methylation levels were obtained from a whole blood methylation study[2] with GEO accession number GSE35069. The study includes DNA methylation levels of six samples from seven purified blood cell subpopulations—CD4+ T cells, CD8+ T cells, CD14+ monocytes, CD19+ B cells, CD56+ NK cells, neutrophils, and eosinophils. For each cell types, we removed the CpG sites whose standard deviations of the six samples exceeded 0.05; for the remaining g_s of G CpG sites, we calculated η_{ag} by computing the median of methylation profiles across six samples.

*Corresponding author: ywei@cuhk.edu.hk

S3 Real Data Preprocessing

We downloaded the rheumatoid arthritis (RA) dataset with the accession number GSE42861 [3] and the GALA II blood methylation data set with accession number GSE77716 [4] from the GEO. For the RA dataset, we removed two samples GSM1051535 and GSM1051691 since their smoking histories were lacking; and for the GALA II blood dataset, the sample GSM2057284 was removed because of lacking gender information. Following [5], CpG sites with a mean methylation level higher than 0.9 or lower than 0.1 were removed. As a result, there are 32,673 and 24,175 CpG sites left for the RA dataset and the GALA II blood methylation dataset, respectively. The beta values of the GALA II blood methylation dataset were normalised by SWAN[6]. In order to compare to the analysis results of previous methods, FineDMR was applied to the adjusted values of both datasets for which the batch effects had been corrected by COMBAT[7].

S4 Details of GEM Algorithm

Let $O_i(t_g), \boldsymbol{\Phi}(t_g)$ and $Y_{ik}(t_g)$ denote the observed aggregated-level DNA methylation value, the evaluation of the basis of the B-splines and the missing data at CpG site g. Collecting $\boldsymbol{Y}_i = [Y_{i1}(t_1), ..., Y_{iK}(t_1), ..., Y_{i1}(t_G), ..., Y_{iK}(t_G)]^T$, with $\widetilde{\boldsymbol{X}}_i = (1, \boldsymbol{X}_i^T)^T$ and $\boldsymbol{P}_i = \text{diag}(\boldsymbol{p}_i^T, G)$, the log transformation of the posterior distribution becomes:

$$
\begin{aligned}
&\log p(\boldsymbol{\Omega} \mid \boldsymbol{\mathcal{W}}, \boldsymbol{Y}) = \sum_i^n logP(\boldsymbol{O}_i \mid \boldsymbol{X}_i, \boldsymbol{Y}_i, \boldsymbol{\Omega}) \\
&+ \sum_i^n logP(\boldsymbol{Y}_i \mid \boldsymbol{\Omega}) + logP(\boldsymbol{\Omega}) \\
&= \sum_{i=1}^n \sum_{g=1}^G \sum_{k=1}^K \{-\frac{1}{2} log(\sigma_\epsilon^2) - \frac{1}{2} \frac{(Y_{ik}(t_g) - \widetilde{\boldsymbol{X}}_i^T \boldsymbol{C}_k \boldsymbol{\Phi}(t_g))^2}{\sigma_\epsilon^2}\} \\
&+ \sum_{i=1}^n \{-\frac{1}{2} log(\mid \boldsymbol{\Psi} \mid) - \frac{1}{2} (\boldsymbol{O}_i - \boldsymbol{P}_i \boldsymbol{Y}_i)^T \boldsymbol{\Psi}^{-1} (\boldsymbol{O}_i - \boldsymbol{P}_i \boldsymbol{Y}_i)\} \\
&- [(\alpha_w + 1) log(w) + \frac{\alpha_w}{\mu_w w} + log(v) + \frac{(log(v) - \mu_v)^2}{2\sigma_v^2}] \\
&- (\alpha_{\sigma^2} + 1) log(\sigma_\epsilon^2) - \frac{\beta_{\sigma^2}}{\sigma_\epsilon^2} + const
\end{aligned}
$$

where $\boldsymbol{\Psi}(i, j) = S(t_i, t_j, v, w)$.

S4.0.1 E-step

In the E-step, the Q function $Q(\boldsymbol{\Omega} \mid \boldsymbol{\Omega}^{(t)}) = \text{E}(\log p(\boldsymbol{\Omega} \mid \boldsymbol{\mathcal{W}}, \boldsymbol{Y}) \mid \boldsymbol{\mathcal{W}}; \boldsymbol{\Omega}^{(t)})$. To compute the Q function, we need to calculate the conditional expectations based on the observed data $\text{E}_{\boldsymbol{Y}}((Y_{ik}(t_g) - \widetilde{\boldsymbol{X}}_i^T \boldsymbol{C}_k \boldsymbol{\Phi}(t_g))^2 \mid \boldsymbol{\mathcal{W}}; \boldsymbol{\Omega})$ for $i = 1, ...n$ and $\text{E}_{\boldsymbol{Y}}((\boldsymbol{O}_i - \boldsymbol{P}_i \boldsymbol{Y}_i)^T \boldsymbol{\Psi}^{-1} (\boldsymbol{O}_i - \boldsymbol{P}_i \boldsymbol{Y}_i) \mid \boldsymbol{\mathcal{W}}; \boldsymbol{\Omega}), i = 1, ...n, g = 1, ..., G$. Therefore, we first derive $P(\boldsymbol{Y}_i \mid \boldsymbol{\mathcal{W}}, \boldsymbol{\Omega})$. Since the observations of different subjects are independent, we can derive $P(\boldsymbol{Y}_i \mid \boldsymbol{\mathcal{W}}, \boldsymbol{\Omega})$s

separately:

$$\begin{aligned}
&-2*\log P(\boldsymbol{Y}_i \mid \boldsymbol{\mathcal{W}}, \boldsymbol{\Omega}) = -(\log P(\boldsymbol{Y}_i \mid \boldsymbol{\Omega}) \\
&+\log P(\boldsymbol{\mathcal{W}} \mid \boldsymbol{\tau}_i, \boldsymbol{\Omega}) - \log(\boldsymbol{\mathcal{W}} \mid \boldsymbol{\Omega})) \\
&= \boldsymbol{Y}_i^T \boldsymbol{P}_i^T \boldsymbol{\Psi}^{-1} \boldsymbol{P}_i \boldsymbol{Y}_i - 2\boldsymbol{O}_i^T \boldsymbol{\Psi}^{-1} \boldsymbol{P}_i \boldsymbol{Y}_i \\
&+\sum_{g=1}^{G}\sum_{k=1}^{K} \frac{Y_{ik}(t_g)^2 - 2Y_{ik}(t_g)(\widetilde{\boldsymbol{X}}_i^T \boldsymbol{C}_k \boldsymbol{\Phi}(t_g))}{\sigma_\epsilon^2} + Const
\end{aligned}$$

then $\boldsymbol{Y}_i \mid \boldsymbol{\mathcal{W}}, \boldsymbol{\Omega} \sim N(\boldsymbol{\Sigma}_i \boldsymbol{L}_i, \boldsymbol{\Sigma}_i)$ where $\boldsymbol{\Sigma}_i = [\boldsymbol{P}_i^T \boldsymbol{\Psi}^{-1} \boldsymbol{P}_i + \mathrm{diag}(\frac{1}{\sigma_\epsilon^2})]^{-1}$, $\boldsymbol{L}_i = \boldsymbol{P}_i^T \boldsymbol{\Psi}^{-1} \boldsymbol{O}_i +$
$$\begin{bmatrix} \frac{\widetilde{\boldsymbol{X}}_i^T \boldsymbol{C}_1 \boldsymbol{\Phi}(t_1)}{\sigma_\epsilon^2} \\ \vdots \\ \frac{\widetilde{\boldsymbol{X}}_i^T \boldsymbol{C}_K \boldsymbol{\Phi}(t_1)}{\sigma_\epsilon^2} \\ \frac{\widetilde{\boldsymbol{X}}_i^T \boldsymbol{C}_1 \boldsymbol{\Phi}(t_G)}{\sigma_\epsilon^2} \\ \vdots \\ \frac{\widetilde{\boldsymbol{X}}_i^T \boldsymbol{C}_K \boldsymbol{\Phi}(t_G)}{\sigma_\epsilon^2} \end{bmatrix},$$
and $\mathrm{E}(\boldsymbol{Y}_i \boldsymbol{Y}_i^T \mid \boldsymbol{\mathcal{W}}, \boldsymbol{\Omega}) = \boldsymbol{\Sigma}_i + \boldsymbol{\Sigma}_i \boldsymbol{L}_i \boldsymbol{L}_i^T \boldsymbol{\Sigma}_i$. Note that $\boldsymbol{\Sigma}_i$ can be calculated blockwise, since CpG sites in different blocks have long distance between each other and hence their correlations in $\boldsymbol{\Psi}$ can be ignored. As a result,

$$\begin{aligned}
&\mathrm{E}_{\boldsymbol{Y}}((Y_{ik}(t_g) - \widetilde{\boldsymbol{X}}_i^T \boldsymbol{C}_k \boldsymbol{\Phi}(t_g))^2 \mid \boldsymbol{\mathcal{W}}; \boldsymbol{\Omega}) \\
=&\mathrm{E}(Y_{ik}(t_g)^2 \mid \boldsymbol{\mathcal{W}}, \boldsymbol{\Omega}) - 2\mathrm{E}(Y_{ik}(t_g) \mid \boldsymbol{\mathcal{W}}, \boldsymbol{\Omega})(\widetilde{\boldsymbol{X}}_i^T \boldsymbol{C}_k \boldsymbol{\Phi}(t_g)) \\
+&(\widetilde{\boldsymbol{X}}_i^T \boldsymbol{C}_k \boldsymbol{\Phi}(t_g))^2 \mathrm{E}_{\boldsymbol{Y}}((\boldsymbol{O}_i - \boldsymbol{P}_i \boldsymbol{Y}_i)^T \boldsymbol{\Psi}^{-1} (\boldsymbol{O}_i - \boldsymbol{P}_i \boldsymbol{Y}_i) \mid \boldsymbol{\mathcal{W}}; \boldsymbol{\Omega}) \\
=&\mathrm{tr}(\boldsymbol{P}_i^T \boldsymbol{\Psi}^{-1} \boldsymbol{P}_i \boldsymbol{\Sigma}_i) + \boldsymbol{L}_i^T \boldsymbol{\Sigma}_i \boldsymbol{P}_i^T \boldsymbol{\Psi}^{-1} \boldsymbol{P}_i \boldsymbol{\Sigma}_i \boldsymbol{L}_i + \boldsymbol{O}_i^T \boldsymbol{\Psi}^{-1} \boldsymbol{O}_i
\end{aligned}$$

S4.0.2 M-step

(1) Update $\boldsymbol{C}_k$

In the M-step, we first update $\boldsymbol{C}_k^{(t+1)}$ by maximizing $Q(\boldsymbol{\Omega} \mid \boldsymbol{\Omega}^{(t)})$. Ignoring the terms without $\boldsymbol{C}_k^{(t+1)}$, the objective function can be written as:

$$l(\boldsymbol{C} \mid \boldsymbol{\Omega}^{(t)}) = \sum_{i=1}^{n}\sum_{g=1}^{G}\sum_{k=1}^{K} (\mathrm{E}(Y_{ik}(t_g) \mid \boldsymbol{\mathcal{W}}, \boldsymbol{\Omega}) - (\widetilde{\boldsymbol{X}}_i^T \boldsymbol{C}_k \boldsymbol{\Phi}(t_g))^2$$

Let D be the number of basis functions, since $\mathrm{E}(Y_{ik}(t_g) \mid \boldsymbol{\mathcal{W}}, \boldsymbol{\Omega})$ is functional variable, $\mathrm{E}(Y_{ik}(t_g) \mid \boldsymbol{\mathcal{W}}, \boldsymbol{\Omega}) \approx \boldsymbol{A}_{ik}^T \boldsymbol{\Phi}(t_g)$ where $\boldsymbol{\Phi}(t_g)$ is a $D \times 1$ vector containing the basis functions on t_g, and $\boldsymbol{A}_{ik}$ is a $D \times 1$ vector. Then, we can update $\boldsymbol{C}_k$ for each k separately:

$$\begin{aligned}
l(\boldsymbol{C}_k \mid \boldsymbol{\Omega}^{(t)}) &\approx \sum_{i=1}^{n}\sum_{g=1}^{G} (\boldsymbol{A}_{ik}^T \boldsymbol{\Phi}(t_g) - \widetilde{\boldsymbol{X}}_i^T \boldsymbol{C}_k \boldsymbol{\Phi}(t_g))^2 \\
&= \sum_{i=1}^{n} (\boldsymbol{A}_{ik}^T - \widetilde{\boldsymbol{X}}_i^T \boldsymbol{C}_k) \boldsymbol{\Phi}^T \boldsymbol{\Phi} (\boldsymbol{A}_{ik}^T - \widetilde{\boldsymbol{X}}_i^T \boldsymbol{C}_k)^T
\end{aligned}$$

Note $\widetilde{\boldsymbol{X}}_i$ is a $(q+1)\times 1$ vector, $\boldsymbol{C}_k$ is a $(q+1)\times D$ vector, and $\boldsymbol{\Phi} = [\boldsymbol{\Phi}(t_1), ..., \boldsymbol{\Phi}(t_G)]^T$ is a $G \times D$ matrix. Let $\boldsymbol{\Phi}^T\boldsymbol{\Phi} = \boldsymbol{B}^{\frac{1}{2}}\boldsymbol{B}^{\frac{1}{2}}$,

$$l(\boldsymbol{C}_k \mid \boldsymbol{\Omega}^{(t)}) \approx \sum_{i=1}^{n}(\boldsymbol{A}_{ik}^T\boldsymbol{B}^{\frac{1}{2}} - \widetilde{\boldsymbol{X}}_i^T\boldsymbol{C}_k\boldsymbol{B}^{\frac{1}{2}})(\boldsymbol{A}_{ik}^T\boldsymbol{B}^{\frac{1}{2}} - \widetilde{\boldsymbol{X}}_i^T\boldsymbol{C}_k\boldsymbol{B}^{\frac{1}{2}})^T$$

Let $\boldsymbol{A}_k$ be an matrix formed by $\boldsymbol{A}_{ik}$ row-wise, and $\boldsymbol{A}_{ik}$ can be estimated by $\hat{\boldsymbol{A}_{ik}} = (\boldsymbol{\Phi}^T\boldsymbol{\Phi})^{-1}\boldsymbol{\Phi}^T(\mathrm{E}(Y_{ik} \mid \boldsymbol{\mathcal{W}}, \boldsymbol{\Omega}))$ where $\mathrm{E}(Y_{ik} \mid \boldsymbol{\mathcal{W}}, \boldsymbol{\Omega}) = [\mathrm{E}(Y_{ik}(t_1) \mid \boldsymbol{\mathcal{W}}, \boldsymbol{\Omega}), ..., \mathrm{E}(Y_{ik}(t_G) \mid \boldsymbol{\mathcal{W}}, \boldsymbol{\Omega})]^T$ is a $G \times 1$ vector. Then, $l(\boldsymbol{C}_k \mid \boldsymbol{\Omega}^{(t)}) \approx \|\ \boldsymbol{A}\boldsymbol{B}^{\frac{1}{2}} - \widetilde{\boldsymbol{X}}\boldsymbol{C}_k\boldsymbol{B}^{\frac{1}{2}}\ \|^2$. Using least squares: $\hat{\boldsymbol{C}}_k = (\widetilde{\boldsymbol{X}}^T\widetilde{\boldsymbol{X}})^{-1}\widetilde{\boldsymbol{X}}^T\boldsymbol{A}_k$ where $\widetilde{\boldsymbol{X}} = [\widetilde{\boldsymbol{X}}_1, ..., \widetilde{\boldsymbol{X}}_n]$ is a $(q+1)\times n$ matrix.

$\hat{\boldsymbol{A}_{ik}}$ can be further separated into $\hat{\boldsymbol{A}_{ik}} = [\hat{\boldsymbol{A}_{ik1}^T}, ..., \hat{\boldsymbol{A}_{ikR}^T}]^T$ since $\boldsymbol{\Phi}$ can be computed blockwise, where $\hat{\boldsymbol{A}_{ikr}} = (\boldsymbol{\Phi}_r^T\boldsymbol{\Phi}_r)^{-1}\boldsymbol{\Phi}_r^T\mathrm{E}(Y_{ikr} \mid \boldsymbol{\mathcal{W}}, \boldsymbol{\Omega})$, $\boldsymbol{\Phi}_r$ is the sub-matrix of $\boldsymbol{\Phi}$ corresponding to block r and $\mathrm{E}(Y_{ikr} \mid \boldsymbol{\mathcal{W}}, \boldsymbol{\Omega}))$ is the sub-vector of $E(Y_{ik} \mid \boldsymbol{\mathcal{W}}, \boldsymbol{\Omega}))$ corresponding to block r. Then $\hat{\hat{\boldsymbol{C}}}_k$ can be computed blockwise.

(2) Update $\boldsymbol{p}_i$

Secondly, we update $\boldsymbol{p}_i^{(t+1)}$ by maximizing $Q(\boldsymbol{\Omega} \mid \boldsymbol{\Omega}^{(t)})$ given $\boldsymbol{C}_k = \boldsymbol{C}_k^{(t+1)}, k = 1, ..., K$. Let $\tilde{\boldsymbol{P}}_i = [\mathrm{diag}(p_{i1}, G), ..., \mathrm{diag}(p_{iK}, G)]$, $\tilde{\boldsymbol{Y}}_i = [Y_{i1}(t_1), ..., Y_{i1}(t_G), ..., Y_{iK}(t_G)]^T$ and $\tilde{\boldsymbol{\Sigma}}_i$ and $\tilde{\boldsymbol{\mu}}_i$ are the corresponding conditional variance-covariance matrix and expectation obtained from E-step. Note that $\tilde{\boldsymbol{P}}_i$ and $\tilde{\boldsymbol{Y}}_i$ have the same elements but different orders compared to $\boldsymbol{P}_i$ and $\boldsymbol{Y}_i$. The objective function is:

$$\begin{aligned}
&l(\boldsymbol{p}_i \mid \boldsymbol{\Omega}^{(t)}, \boldsymbol{C}^{(t+1)}) = \mathrm{E}_{\boldsymbol{Y}}((\boldsymbol{O}_i - \boldsymbol{P}_i\boldsymbol{Y}_i)^T\boldsymbol{\Psi}^{-1}(\boldsymbol{O}_i - \boldsymbol{P}_i\boldsymbol{Y}_i) \mid \boldsymbol{\mathcal{W}}; \boldsymbol{\Omega})\\
&= \mathrm{E}_{\boldsymbol{Y}}((\boldsymbol{O}_i - \tilde{\boldsymbol{P}}_i\tilde{\boldsymbol{Y}}_i)^T\boldsymbol{\Psi}^{-1}(\boldsymbol{O}_i - \tilde{\boldsymbol{P}}_i\tilde{\boldsymbol{Y}}_i) \mid \boldsymbol{\mathcal{W}}; \boldsymbol{\Omega})\\
&= \mathrm{tr}(\tilde{\boldsymbol{P}}_i^T\boldsymbol{\Psi}^{-1}\tilde{\boldsymbol{P}}_i\tilde{\boldsymbol{\Sigma}}_i) + \tilde{\boldsymbol{\mu}}_i^T\tilde{\boldsymbol{P}}_i^T\boldsymbol{\Psi}^{-1}\tilde{\boldsymbol{P}}_i\tilde{\boldsymbol{\mu}}_i - 2\boldsymbol{O}_i^T\boldsymbol{\Psi}^{-1}\tilde{\boldsymbol{P}}_i\boldsymbol{Y}_i
\end{aligned}$$

subject to $\sum_{k=1}^{K} p_{ik} = 1$. This problem can be solved by quadratic programming.

(3) Update σ_ϵ^2

Thirdly, we update σ_ϵ^2 by maximizing $Q(\boldsymbol{\Omega} \mid \boldsymbol{\Omega}^{(t)})$ given $\boldsymbol{C}_k = \boldsymbol{C}_k^{(t+1)}, k = 1, ..., K$ and $\boldsymbol{p}_i = \boldsymbol{p}_i^{(t+1)}, i = 1, ..., n$. The objective function is:

$$\begin{aligned}
l(\sigma_\epsilon^2 \mid \boldsymbol{\Omega}^{(t)}, \boldsymbol{C}^{(t+1)}, \boldsymbol{p}^{(t+1)}) = \sum_{i=1}^{n}\sum_{g=1}^{G}\sum_{k=1}^{K}\{-\frac{1}{2}log(\sigma_\epsilon^2)\\
- \frac{1}{2}\frac{\mathrm{E}_{\boldsymbol{Y}}((Y_{ik}(t_g) - \widetilde{\boldsymbol{X}}_i^T\boldsymbol{C}_k\boldsymbol{\Phi}(t_g))^2 \mid \boldsymbol{\mathcal{W}}; \boldsymbol{\Omega})}{\sigma_\epsilon^2}\}
\end{aligned}$$

This problem has an analytical solution.

(4) Update $\boldsymbol{\theta}$

Finally, we update $\boldsymbol{\theta}^{(t+1)}$ by maximizing $Q(\boldsymbol{\Omega} \mid \boldsymbol{\Omega}^{(t)})$ given $\boldsymbol{C}_k = \boldsymbol{C}_k^{(t+1)}, k = 1, ..., K$, $\boldsymbol{p}_i = \boldsymbol{p}_i^{(t+1)}, i = 1, ..., n$ and $\sigma_\epsilon^2 = [\sigma_\epsilon^2]^{(t+1)}$. We use gradient descent to find the optimizer $\boldsymbol{\theta}^{(t+1)}$. The objective function is:

$$\begin{aligned}
&l(\boldsymbol{\theta} \mid \boldsymbol{\Omega}^{(t)}) = \sum_{i=1}^{n} -\frac{1}{2}log(\mid \boldsymbol{\Psi} \mid) - \frac{1}{2}[\mathrm{tr}(\boldsymbol{P}_i^T\boldsymbol{\Psi}^{-1}\boldsymbol{P}_i\boldsymbol{\Sigma}_i) + \boldsymbol{O}_i^T\boldsymbol{\Psi}^{-1}\boldsymbol{O}_i\\
&+ \boldsymbol{L}_i^T\boldsymbol{\Sigma}_i\boldsymbol{P}_i^T\boldsymbol{\Psi}^{-1}\boldsymbol{P}_i\boldsymbol{\Sigma}_i\boldsymbol{L}_i - 2\boldsymbol{O}_i^T\boldsymbol{\Psi}^{-1}\boldsymbol{P}_i\boldsymbol{\Sigma}_i\boldsymbol{L}_i]\\
&- (\alpha_w + 1)log(w) - \frac{\alpha_w}{\mu_w w} - log(v) - \frac{(log(v) - \mu_v)^2}{2\sigma_v^2}
\end{aligned}$$

References

[1] Kiselev, V., Yiu, A. & Hemberg, M. scmap: projection of single-cell RNA-seq data across data sets. *Nature Methods.* **15**, 359-362 (2018)

[2] Reinius, L., Acevedo, N., Joerink, M., Pershagen, G., Dahlén, S., Greco, D., Söderhäll, C., Scheynius, A. & Kere, J. Differential DNA methylation in purified human blood cells: implications for cell lineage and studies on disease susceptibility. *PloS One.* **7**, e41361 (2012)

[3] Liu, Y., Aryee, M., Padyukov, L., Fallin, M., Hesselberg, E., Runarsson, A., Reinius, L., Acevedo, N., Taub, M., Ronninger, M. & Others Epigenome-wide association data implicate DNA methylation as an intermediary of genetic risk in rheumatoid arthritis. *Nature Biotechnology.* **31**, 142-147 (2013)

[4] Pino-Yanes, M., Thakur, N., Gignoux, C., Galanter, J., Roth, L., Eng, C., Nishimura, K., Oh, S., Vora, H., Huntsman, S. & Others Genetic ancestry influences asthma susceptibility and lung function among Latinos. *Journal Of Allergy And Clinical Immunology.* **135**, 228-235 (2015)

[5] Zou, J., Lippert, C., Heckerman, D., Aryee, M. & Listgarten, J. Epigenome-wide association studies without the need for cell-type composition. *Nature Methods.* **11**, 309-311 (2014)

[6] Maksimovic, J., Gordon, L. & Oshlack, A. SWAN: Subset-quantile within array normalization for illumina infinium HumanMethylation450 BeadChips. *Genome Biology.* **13** pp. 1-12 (2012)

[7] Johnson, W., Li, C. & Rabinovic, A. Adjusting batch effects in microarray expression data using empirical Bayes methods. *Biostatistics.* **8**, 118-127 (2007)

Table S1: Effects of degree of freedom for the B-Spline on the performance of FineDMR in the true alternative case with n=600.

Degree of freedom	$blocksize/10 + 3$	$blocksize/15 + 3$	$blocksize/5 + 3$
TPR(aggrgated)	0.889	0.919	0.841
FPR(aggrgated)	0.003	0.01	0.0006
TPR(cell-type-specific)	0.834	0.904	0.819
FPR(cell-type-specific)	0.0005	0.003	0.00001
